# Arsenic Exposure Increases Monocyte Adhesion to the Vascular Endothelium, a Pro-Atherogenic Mechanism

**DOI:** 10.1371/journal.pone.0136592

**Published:** 2015-09-02

**Authors:** Maryse Lemaire, Luis Fernando Negro Silva, Catherine A. Lemarié, Alicia M. Bolt, Manuel Flores Molina, Regina M. Krohn, Judit E. Smits, Stéphanie Lehoux, Koren K. Mann

**Affiliations:** 1 Department of Oncology, Lady Davis Institute for Medical Research, McGill University, Montréal, Québec, Canada; 2 Division of Experimental Medicine, Department of Medicine, Lady Davis Institute for Medical Research, McGill University, Montréal, Québec, Canada; 3 Department of Medicine, Lady Davis Institute for Medical Research, McGill University, Montréal, Québec, Canada; 4 Department of Ecosystem and Public Health, Faculty of Veterinary Medicine, University of Calgary, Calgary, Alberta, Canada; Maastricht University Faculty of Health, Medicine, and Life Sciences, NETHERLANDS

## Abstract

Epidemiological studies have shown that arsenic exposure increases atherosclerosis, but the mechanisms underlying this relationship are unknown. Monocytes, macrophages and platelets play an important role in the initiation of atherosclerosis. Circulating monocytes and macrophages bind to the activated vascular endothelium and migrate into the sub-endothelium, where they become lipid-laden foam cells. This process can be facilitated by platelets, which favour monocyte recruitment to the lesion. Thus, we assessed the effects of low-to-moderate arsenic exposure on monocyte adhesion to endothelial cells, platelet activation and platelet-monocyte interactions. We observed that arsenic induces human monocyte adhesion to endothelial cells *in vitro*. These findings were confirmed *ex vivo* using a murine organ culture system at concentrations as low as 10 ppb. We found that both cell types need to be exposed to arsenic to maximize monocyte adhesion to the endothelium. This adhesion process is specific to monocyte/endothelium interactions. Hence, no effect of arsenic on platelet activation or platelet/leukocyte interaction was observed. We found that arsenic increases adhesion of mononuclear cells via increased CD29 binding to VCAM-1, an adhesion molecule found on activated endothelial cells. Similar results were observed *in vivo*, where arsenic-exposed mice exhibit increased VCAM-1 expression on endothelial cells and increased CD29 on circulating monocytes. Interestingly, expression of adhesion molecules and increased binding can be inhibited by antioxidants *in vitro* and *in vivo*. Together, these data suggest that arsenic might enhance atherosclerosis by increasing monocyte adhesion to endothelial cells, a process that is inhibited by antioxidants.

## Introduction

Arsenic is a tasteless and odorless environmental pollutant to which millions of people worldwide are exposed, mainly through consumption of contaminated food and water [1,2]. The World Health Organization, the United States Environmental Protection Agency and Health Canada have set the maximum contamination level at 10 ppb in the municipal water [3], but well water in many areas can contain higher levels [2,4]. For instance, over 10% of wells analyzed in Nova Scotia (Canada) have more than 500 ppb arsenic [5], and high endemic areas with levels up to 2.5 **ppm** are found in Bangladesh, the southwestern United States and Taiwan [2]. Growing epidemiologic evidence indicates that individuals exposed to arsenic, even at low concentrations, have an increased risk of developing cardiovascular diseases, including atherosclerosis [6,7,8]. Moreover, we and others have demonstrated that arsenic induces atheroma formation in apolipoprotein E-deficient (apoE^-/-^) mouse models of atherosclerosis [9,10,11]. Interestingly, arsenic increased atherosclerosis without concomitant changes in circulating lipid profiles that could have contributed to the enhanced pathology [9]. Thus, the mechanisms by which arsenic exerts its effects remain uncertain.

Atherosclerosis is a multi-factorial disease resulting from a series of specific cellular and molecular events. Initiation of atherosclerosis involves endothelial cell activation by various stimuli, including cytokines, high levels of reactive oxygen species (ROS), and oxidized low density lipoprotein [12]. Arsenic has been described to participate in endothelial cell activation. In cultured endothelial cells, arsenic increases inflammatory molecule production [13], ROS [14,15] and uptake of oxidized lipids [16], all of which have been linked to atherosclerosis. Once activated, the endothelial cells express adhesion molecules, including vascular cell adhesion molecule 1 (VCAM-1; CD106). VCAM-1 allows circulating mononuclear cells to firmly adhere to the endothelium and to transmigrate into the vascular wall [17,18], and subsequent endothelial cell signaling generates low levels of ROS to support monocyte diapedesis [19]. In the arterial intima, monocytes differentiate to macrophages, engulf oxidized lipids, become lipid laden, and form foam cells, which contribute to the atherosclerotic core formation [20]. Therefore, monocyte binding to VCAM-1 is an essential step in atheroma formation. This process is facilitated by VCAM-1 binding to the *very late activation antigen-4* ligand (VLA-4; CD49d/CD29; hereafter called CD29) expressed by leukocytes. VLA-4 is an integrin receptor of the β1 family that selectively binds to VCAM-1 [21].

Although arsenic-mediated pro-atherogenic effects on macrophages have been described, including impaired cytokine secretion and immune responses [22,23], little is known about monocytes as cellular targets for arsenic. Similarly, neutrophils are found along the aortic vascular wall during atherosclerosis in apoE^-/-^ mouse model of atherosclerosis [24], and activated platelets are reported to increase monocyte adhesion to the vascular lesions and enhance plaque formation [25], but the effects of arsenic on these cells remains unknown.

Here, we hypothesized that arsenic may affect early events in atherogenesis. Thus, we assessed the effects of a low-to-moderate arsenic exposure on monocyte, neutrophil, platelet, and endothelial cell interactions as potential pro-atherogenic mechanisms.

## Materials and Methods

### Chemicals

We utilized two trivalent inorganic arsenic compounds for these studies. To compare with established literature, arsenic trioxide (FW 197.84 g/L; As_2_O_3_) (Sigma-Aldrich, Oakville, Ontario, Canada) was used for all the *in vitro* assays. When dissolved in NaOH, arsenic trioxide will form arsenite (Balanced equation: As_2_O_3_ + 2 NaOH = H_2_O + 2 NaAsO_2_) [26]. Therefore, it was dissolved in 0.1 N NaOH and subsequently diluted in sterile phosphate buffer saline solution (PBS) prior addition to the cells. However, because of its greater dissolution index in water, *m*-sodium arsenite (FW 129.91 g/L; NaAsO_2_) (Sigma-Aldrich) was used as a source of arsenic for the *in vivo* exposures [26]. Thus, we used the *ppb* nomenclature to compare the concentration of the arsenic molecules in solution from these two sources of arsenic. The antioxidant N-acetylcysteine (NAC) is from Sigma-Aldrich.

### Cell culture

Human monocytic U937 cells (ATCC CRL-1593.2; Manassas, Virginia, USA) and human peripheral blood primary monocytes were cultured in RPMI-1640 medium (Invitrogen Inc., Ontario, Canada). Human peripheral blood mononuclear cells (PBMC) were differentiated into macrophages with macrophage colony stimulating factor (M-CSF; 50 ng/ml; PeproTech, NJ, USA) for 12 days. Human cells were obtained after participants provided their written informed consent using a protocol approved by the Research Ethics Review Board (REB) of the Jewish General Hospital. Murine bone marrow primary monocytes were cultured in RPMI-1640 medium containing 5% β-mercaptoethanol (Sigma-Aldrich). Human umbilical vein/vascular endothelium (HUVEC) cells were kindly provided by Dr. Mark Blostein (Lady Davis Institute for Medical Research, Montréal, Qc, Canada), who acquired those cells from ATCC (CRL-1730), and were sustained in F-12K medium (ATCC) containing 0.1 mg/ml heparin (Sigma-Aldrich), 0.03 mg/ml endothelial cell growth supplement (AbD Serotec, Raleigh, NC, USA) on 0.1% gelatin-coated plates. All cells were cultured in medium containing 10% fetal bovine serum (FBS; Wisent, St-Bruno, Qc, Canada) and penicillin/streptomycin (Wisent) at 37°C with 5% CO_2_.

### Animals

Wild-type C57BL/6 and B6.129P2-*apoe* ^*tm1Unc*^/J (apoE^-/-^) male mice were obtained from Jackson laboratory (Bar Harbor, Maine, USA). The McGill Animal Care Committee approved the experimental protocol and animals were handled in accordance with institutional guidelines, which followed the Canadian Council of Animal Care. The McGill Animal Care Committee is IACUC approved. For long-term arsenic exposure experiments, apoE^-/-^ male mice (3 week old, n = 5 animals per group) were grouped with cage companionsand fed *ad libitum* with normal rodent chow (2018; Harlan Laboratories Inc., WI, USA)or deficient (0.009 mg/kg) or high (0.3 mg/kg) selenium-containing lentil diet (Krohn *et al*, [27] *in press*) at the animal facility. The normal rodent chow contains 0.2 mg/kg selenium (2018; Harlan Laboratories). Low levels of arsenic were detected both in the tap water (0.75 ppb) and in the normal rodent chow (1.90 ppb) ± S.D. of 5%, with detection limit of 0.65 ppb [9]. Starting at 5 weeks of age, mice were either maintained on tap water or on tap water-containing 200 ppb arsenic (0.35 mg/L NaAsO_2_) for 8 or 13 weeks, as described previously [9]. We decided to feed the animals with selenium-containing food instead of adding the selenium in the water. This prevents formation of arsenic-selenium complexes in the water. As we have previously described [9], no obvious toxicities were observed in mice given arsenic at any time during the experiment.

### Plasma analyses

Blood (0.6 ml) was collected using EDTA-coated tubes (Sarstedt, Germany) by cardiac puncture and plasma was recuperated. Circulating levels of the chemokine CCL5 (RANTES) and CCL2 (MCP-1) were measured using an immunoassay kit (multiplex bead-based) on a Bio-Plex 200 (Bio-Rad Laboratories, ON, Canada), as previously described [9]. Each sample (*n* ≥ 4 animals per group) was analyzed in duplicate.

### Isolation of primary human and murine cells

In order to obtain human primary monocytes, blood samples (50–100 ml) were collected from healthy normal donors in tubes coated with sodium heparin (BD Vacutainer). These cells were obtained after participants provided their written informed consent using a protocol approved by the REB of the Jewish General Hospital. The REB also approved the procedures. Samples were centrifuged for 10 min at 1200 rpm to separate the plasma from the cells. Cells were diluted in HBSS medium (Wisent), slowly layered onto a Ficoll solution (GE Healthcare Life Sciences, Baie d’Urfé, Qc, Canada) and centrifuged at 2200 rpm for 30 min. The medium layer containing mononuclear cells was collected and diluted in HBSS medium. Cells were centrifuged again for 10 min at 1200 rpm and the pellets were collected and washed in HBSS. Peripheral blood mononuclear cells were seeded in RPMI-1640 + 10% FBS and allowed to adhere to plastic for 1.5 h in order to enrich the monocytic population [28]. The supernatant containing non-adherent cells was removed and fresh RPMI-1640 + 10% FBS was added to the attached monocytes.

To isolate wild-type murine monocytes from bone marrow, tibiae from C57BL/6 mice were flushed with RPMI-1640 medium. Cells were homogenized with 18G needle, centrifuged and suspended in RPMI-1640 + 10% FBS in single cell suspension prior to performing monocyte-enriching adherence step.

### Platelets preparation, measurement of platelets activation and assessment of platelets aggregates with neutrophil or monocytes

To study the effects of arsenic exposure on platelet activation in context of atherosclerosis, we collected platelets from C57Bl/6 and apoE^-/-^ mice. Blood samples were taken from the saphenous vein using EDTA-coated tubes (Sarstedt, Germany). Blood was diluted 1:1 with flow cytometry buffer, consisting of PBS supplemented with 5% FBS and 0.01M sodium azide, and centrifuged at 60 g for 10 min to recuperate the platelet rich plasma (PRP). PRP was further centrifuged at 240 g for 10 min, and the pellet was suspended in flow cytometry buffer.

In order to evaluate platelet activation, 5 x 10^6^ platelets were seeded on fibrinogen-coated (30 ug/mL) coverslips in wash buffer [150 mM NaCl, 20 mM PIPES (Sigma-Aldrich), pH 6.5]. Platelets were then exposed for 5 min to arsenic or human α-thrombin, a platelet activator (1U, Haematologic Technologies Inc, Essex Junction, Vermont, USA). The thrombin was aliquoted in sterile PBS, kept at -20°C, and used fresh for every experiment. Once rinsed, PRP was exposed to 0.1% Triton X-100 (Amresco, Solon, Ohio, USA) at room temperature in the dark for 1 h. Triton was removed, and PRP was blocked with BSA/PBS 0.1% Tween 20 (Bio-Rad Laboratories) for 1 h. Blocking media was removed, and PRP was stained with fluorescent dye (Phalloidin; Alexa Fluor; Molecular Probes; Life Technologies, Burlington, ON, Canada) for 2 h in the dark. Images for platelet spreading were acquired using *Infinity Capture* software and camera (Lumenera, Canada). Assessment of surface platelet activation biomarker was also performed. 5 x 10^6^ platelets were centrifuged at 1000 rpm for 5 min. Arsenic and thrombin were added directly to the pellet, for 5 min. After incubation, platelets were washed with flow cytometry buffer (0.5 ml) prior to assess cellular surface biomarkers of activation, as described below.

In addition to platelet activation assays, platelet/monocyte and platelet/ neutrophil aggregates were detected *in vivo* in order to evaluate the effects of arsenic exposure on their formation. CD14^+^/CD41^+^ platelet/monocyte and Ly6G^+^/CD41^+^ platelet/neutrophil aggregates were monitored at 14, 21 and 28, or 21, 28 and 35 days of arsenic exposure, respectively. Blood was collected as for platelet activation assays, and surface markers detected as described below. One mouse received lipopolysaccharide (LPS; 1 mg/kg; Sigma-Aldrich) for 18 h as a positive control.

### Cellular surface antigen assessment

Surface antigens were detected by direct immunofluorescence using flow cytometry (Beckman Coulter, Mississauga, Ontario, Canada and LSRFortessa Cell Analyzer (BD Biosciences, San Jose, California, USA). Briefly, control cells and cells exposed to arsenic were washed twice with PBS supplemented with 5% FBS (Wisent) and 0.01 M sodium azide (flow cytometry buffer). The cells were then exposed to labeled anti-CD29 (eBioscience anti-murine; Pharmingen anti-human: HUTS-21 clone), P-selectin (BD Pharmingen), CD14 (eBioscience), CD41 (BD Pharmingen), Ly6G (eBioscience) or their specific isotype control antibody and incubated for 45 min on ice in the dark. Cells were then washed twice with flow cytometry buffer, fixed in 2% paraformaldehyde and analyzed by flow cytometry. The gates for positive-staining cells were determined by comparison with cells stained with the isotype-matched control antibodies. The FCS express and FlowJo softwares (denovosoftware, CA, USA; flowJo LLC, Ashland, Oregon, USA) were used to analyze the data.

### Cell adhesion assay

Cell culture plates were coated overnight with recombinant mouse VCAM-1/Fc chimera (2 μg/mL; R&D Systems; MN, USA) at 4°C, rinsed with PBS and saturated with 2% BSA for 1 h. Human and murine primary circulating monocytes or U937 cells, were incubated with CellTracker orange CMTMR fluorescent dye (0.25 μl/ml; Molecular Probes; Life Technologies) in RPMI-1640 for 30 min, centrifuged and resuspended in RPMI-1640 + 10% FBS culture medium. Fluorescently-labelled cells (1000 cells/well) were incubated with the VCAM-1/Fc chimera-coated plates for 30 min with or without CD29 blocking antibody (0.5 μg; BD Pharmingen, clone 9EG7). Non-adherent cells were washed twice with PBS, and the adherent fluorescent cells were counted under a fluorescence microscope.

To assess monocyte adhesion to endothelial cells, HUVEC cells were placed on 0.1% gelatin-coated cover slides. At 80% confluence, cells were exposed to arsenic for 72 h. Arsenic-exposed, fluorescently-labelled U937 cells (1000 cells/ml) were seeded over HUVEC and allowed to adhere for 1 h. The non-adherent cells were washed away, and the co-culture was stained with DAPI 1:2 into mounting media (Vectashield h-2000, Vector Laboratories: Immu-Mount, Thermo Scientific, respectively) and the adherent fluorescent cells were counted under a fluorescence microscope and expressed relative to the total number of cells per field.

### Organ culture

Bone marrow and carotids were isolated from C57BL/6 12-week-old male mice. The carotids were connected to a perfusion circuit consisting of a 3-port reservoir, a pump and a pressure chamber, as previously described [29,30]. The arteries were immersed in the chamber in DMEM (Invitrogen Inc) + 5% FBS culture medium, and the circulation initiated overnight with medium with and without arsenic. In parallel, the isolated bone marrow cells were also cultured overnight in RPMI-1640 with 10% FBS with and without arsenic. These cells were then fluorescently-labelled (CellTracker orange CMTMR; Life technologies) and injected in the carotids [31]. They were allowed to adhere to the endothelial cells of the vasculature wall for 30 min before being washed. In some experiments, CD29 blocking antibody (0.5 μg) was added for 30 min prior to the bone marrow cell injection in the system. Total adherent cells were counted under a fluorescent microscope and results are expressed relative to surface area (number of cells / [2πrh + 2(πr^2^)] * 10^6^; r = radius, h = length of the vessel).

### Immunohistochemical analysis

ApoE^-/-^ murine carotids arteries were removed (n = 4), rinsed, fixed in 4% paraformaldehyde and incubated overnight in a 30% sucrose solution, as previously described [9]. Carotids were then frozen in Tissue Tek OCT (Sakura, CA) compound, and serial cryosections of 6-μm thickness were sliced. Carotids were stained with primary antibodies against VCAM-1 (sc-8304; Santa Cruz Biotechnology, Dallas, TX, USA). Briefly, four to six sections/animal were incubated with primary anti-VCAM-1 antibodies (1:200) at room temperature. Biotinylated-secondary antibody (1:500; BA-1000; Vector Laboratories, Burlingame, California, USA) was incubated at room temperature for 30 minutes, then slides were processed using the peroxidase VECTASTAIN ABC kit (Vector Laboratories) and developed with ImmPACT DAB peroxidase substrate (Vector Laboratories). Sections were counterstained with 2% Harris modified hematoxylin (Thermo Fisher Scientific, Waltham, Maine, USA) and mounted in Permount (Thermo Fisher Scientific). Images were acquired using *Infinity Capture* software and camera (Lumenera). VCAM-1 is expressed as the number of positive cells relative to the carotid luminal perimeter using ImageJ software (NIH).

### Detection of superoxide

U937 cells were pre-treated with the antioxidant NAC (1 mM) for 1 h. Media was changed, and the cells (1 x 10^6^ cells) were exposed to arsenic or vehicle control for 3 h. Cells were then stained with 2 μM hydroethidine (HEt; Molecular Probes) in PBS supplemented with 1% FBS for 30 min at 37°C. Cells were washed with warm PBS, and analyzed by flow cytometry (Beckman Coulter).

Carotids from ApoE^-/-^ mice fed either deficient or high-selenium food pellets were removed, fixed, frozen and sliced as previously described [9]. Het (0.1 mM) diluted in PBS was added directly on the frozen tissue, and pictures were acquired using *Infinity Capture* software and camera (Lumenera, Canada). Positive staining was expressed as percentage of vascular wall area from at least 3 sections per animal (n = 4 animals) using Image J software (NIH).

### Statistical considerations

For statistical analysis, the one-way ANOVA was performed and the p value was evaluated with a Tukey’s post hoc test using the GraphPad Instat software (San Diego, CA, USA). A p value < 0.05 indicates statistical significance. The data correspond to the mean values ± S.D.

## Results

### Arsenic induces monocyte adhesion to endothelial cells, with maximal binding achieved following exposure of both cell types

**Fig 1:**
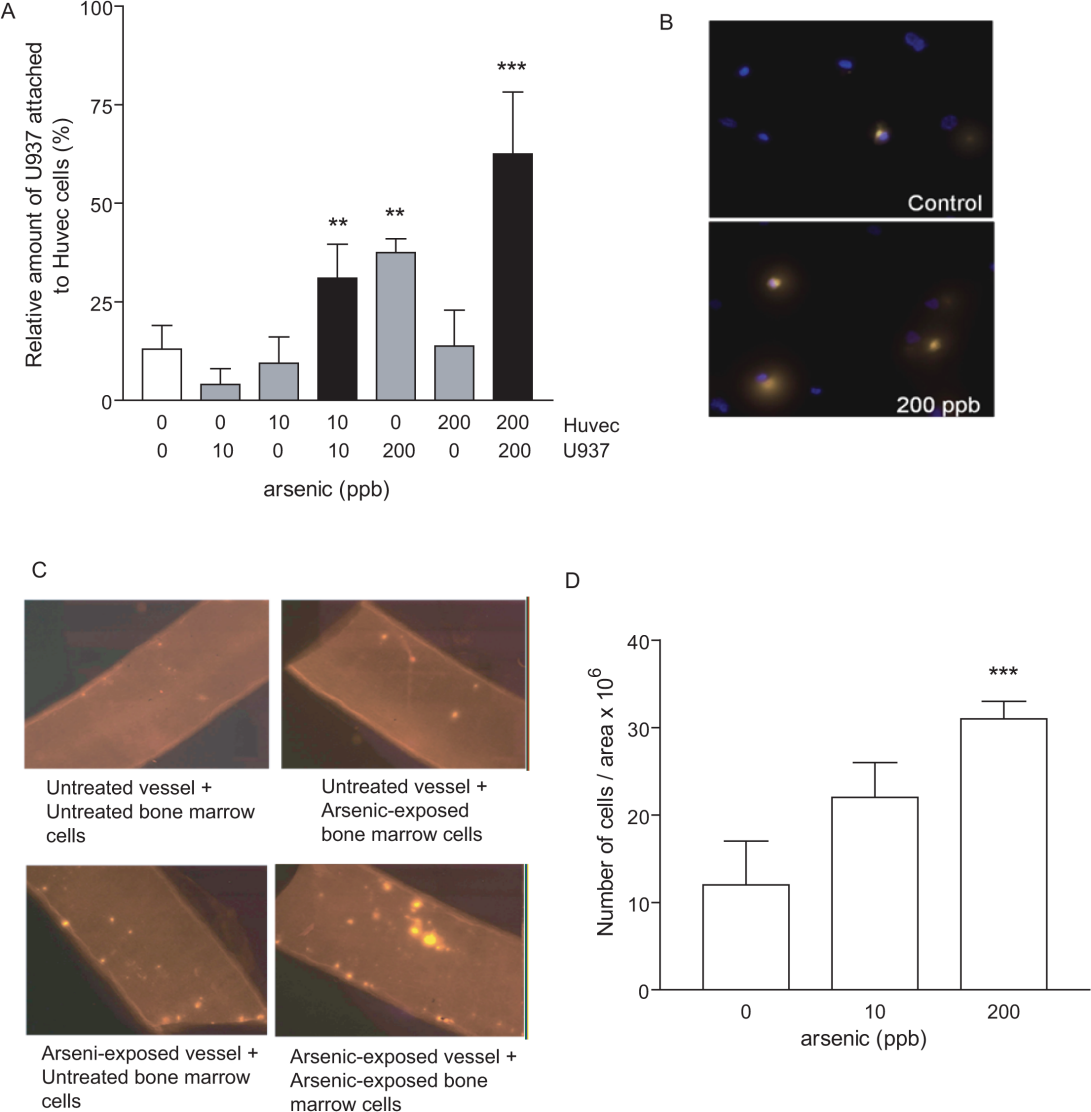
Arsenic induces monocyte adhesion to endothelial cells, with maximal binding achieved following exposure of both cell types. (A) U937 and/or HUVEC cells (1000 cells/ml) were exposed to arsenic overnight (0, 10 or 200 ppb). U937 cells were fluorescently-labelled and were incubated with HUVEC cells. The non-adhered cells were washed away, and the adherent fluorescent cells were counted. Data are expressed as relative number of U937 over total HUVEC stained cells. ** = p<0.01; *** = p<0.001 B) Representative pictures are shown (40X). C-D) Organ culture of carotid arteries and fluorescently-labelled bone marrow cells where neither, one, or both components were exposed to arsenic trioxide overnight and allowed to adhere to each other for 30 min before being washed. Adherent cells were counted. Representative pictures of 200 ppb arsenic-exposed are shown in C. D) Both components were exposed to either 10 or 200 ppb arsenic. Data represent ratio ± S.D., n ≥ 3. **: p < 0.01; ***: p < 0.001, compared to unexposed controls.

The interaction between monocytes and vascular endothelium is one of the initial events in atherosclerotic plaque formation. We hypothesized that arsenic increased this interaction as part of its pro-atherogenic mechanisms. In order to assess the effect of arsenic on monocyte adhesion to endothelial cells, we studied the adhesion *in vitro* and *ex vivo*. First, we assessed binding of U937 monocytic cells to HUVECs, where either cell type or both were exposed to arsenic for 72 hours (10 or 200 ppb). While arsenic exposure of HUVECs alone did not increase adhesion of U937 cells, U937 exposed to 200 ppb arsenic adhered significantly more to untreated HUVECs (Fig 1A and 1B). Interestingly, when both cell types were exposed to arsenic, there was significantly more binding. This was most marked for the lowest concentration of arsenic. While 10 ppb arsenic exposure of either cell type alone did not increase adhesion, it significantly enhanced binding when both cell types were treated (Fig 1A).

Next, we extrapolated these findings to an *ex vivo* model using murine carotids arteries and bone marrow cells [31]. This model allowed us to determine interactions between primary mononuclear cells and vascular endothelium on its native basal lamina. This is particularly important considering data that endothelial cell inflammatory signaling can differ depending on the extracellular matrix used in culture [32]. The vessel, the total bone marrow cells, or both were exposed to arsenic. The fluorescently-labelled, bone marrow cells were subsequently injected into the carotid and allowed to adhere to the vascular endothelium for 30 min. We found that, as with our *in vitro* cultures, exposure of both the endothelium and the bone marrow cells leads to maximal binding (Fig 1C). Furthermore, *ex vivo* adhesion is dose-dependent and is achieved at arsenic concentrations, as low as 10 ppb (Fig 1D) when both components are exposed to arsenic.

### Arsenic does not enhance platelet activation, platelet/monocyte or platelet/ neutrophil interactions

**Fig 2:**
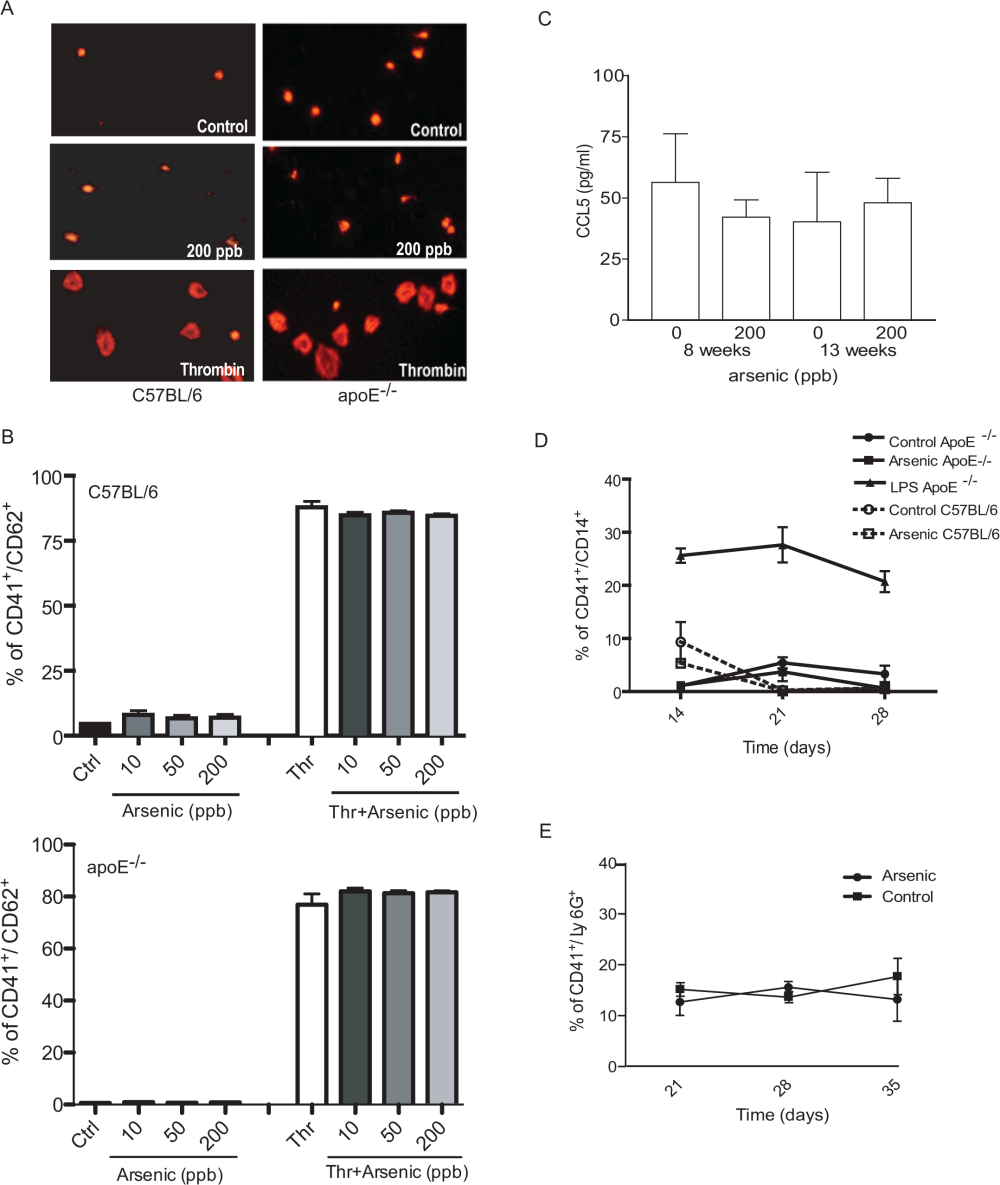
Arsenic does not enhance platelet activation, platelet/monocyte interaction or platelet/neutrophil aggregates formation. C57BL/6 wild-type and apoE^-/-^ mouse platelets were collected and exposed to arsenic (10, 50 or 200 ppb) and/or thrombin for 5 minutes. Representative pictures of platelet spreading after control, 200 ppb arsenic or 1U thrombin are shown in A (left panels: C57BL/6; right panels: apoE^-/-^). (B) P-selectin (CD62) expression was assessed by flow cytometry (up panel: C57BL/6; down panel: apoE^-/-^). (C) Circulating CCL5 levels were measured in apoE^-/-^ male mice were left untreated or exposed to 200 ppb arsenic for 8 or 13 weeks using an immunoassay kit (multiplex bead-based) on a Bio-Plex 200 (Bio-Rad Laboratories, ON, Canada). Each sample (n = 4) was analyzed in duplicate (technical replicate). (D-E) The platelet/monocyte aggregates (CD14^+^/CD41^+^; D) and the platelet/neutrophil aggregates (Ly6G^+^/CD41^+^; E) were followed from day 14 to 28 in the circulation of mice exposed to 200 ppb arsenic. One mouse, as positive control, was treated with LPS for 18 hours before the blood collection. Values are expressed as mean ± S.D., n ≥ 3 animals.

In addition to endothelial cell/monocyte interactions, adhesion of monocytes and neutrophils to circulating platelets contributes to plaque formation [24,25]. Platelets, once activated, induce cytokine secretion from the endothelium to recruit leukocytes to the vasculature. Hence, we assessed the effects of low-to-moderate arsenic exposure on platelet activation, and on platelet/monocyte and platelet/neutrophil aggregation. First, we determined whether arsenic could activate platelets from C57BL/6 or apoE^-/-^ mice, as has been shown in wild-type rats [33]. We were particularly interested in apoE^-/-^ mice, because this hyperlipidemic strain was used in our *in vivo* model of arsenic enhanced atherosclerosis [9]. We measured platelet activation either by spreading morphology or P-selectin expression, a surface marker of platelet activation (reviewed in [34]). In contrast to reported data [33], our results showed that arsenic alone did not activate murine C57Bl/6 or apoE^-/-^ platelets in either assay (Fig 2A and 2B), whereas thrombin, a known platelet agonist, induced platelet spreading and P-selectin expression. However, arsenic did not alter thrombin-induced platelet activation. Additionally, we assessed the circulating levels of CCL5, a cytokine secreted from activated platelets that promotes early recruitment of monocytes and neutrophils to the endothelium [35]. CCL5 levels were assessed in plasma from arsenic-exposed apoE^-/-^ mice after 8 and 13 weeks, time points where significant arsenic-enhanced atherosclerosis is observed [9]. However, CCL5 levels were unchanged by arsenic at the time of the early 8 week lesion and the well-established 13 week lesion (Fig 2C).

Monocyte- and neutrophil-platelet aggregates are recruited to the activated vascular endothelium early in the atherosclerotic process. Thus, we evaluated the circulating platelet/monocyte and platelet/neutrophil aggregates in the whole blood of apoE^-/-^ mice treated over 28 days with 200 ppb arsenic, a concentration known to cause a significant increase in plaque formation [9]. No significant changes were observed over this period in either monocyte or neutrophil aggregation with platelets (Fig 2D and 2E). However, the LPS-exposed mice, used as positive control, had a significantly higher platelet/monocyte aggregate formation (Fig 2D). Together, these data indicate that arsenic specifically enhances the interactions between monocytic cells and the endothelium, but not the interactions of leukocytes with platelets.

Circulating levels of CCL2 (MCP-1) can enhance monocyte/endothelial cell adhesion [36]. Thus, we measured CCL2 in the plasma of apoE^-/-^ mice given 200 ppb arsenic orally for either 8 or 13 weeks. No significant changes in circulating levels of CCL2 in mice exposed to arsenic as compared to tap water controls (S1 Fig). These levels were also not elevated compared to CCL2 levels observed in wild-type C57BL/6 mice. This suggests that circulating CCL2 levels are not increased by arsenic in this model of atherosclerosis.

### Arsenic induces mononuclear cell adhesion to VCAM-1 via CD29

**Fig 3:**
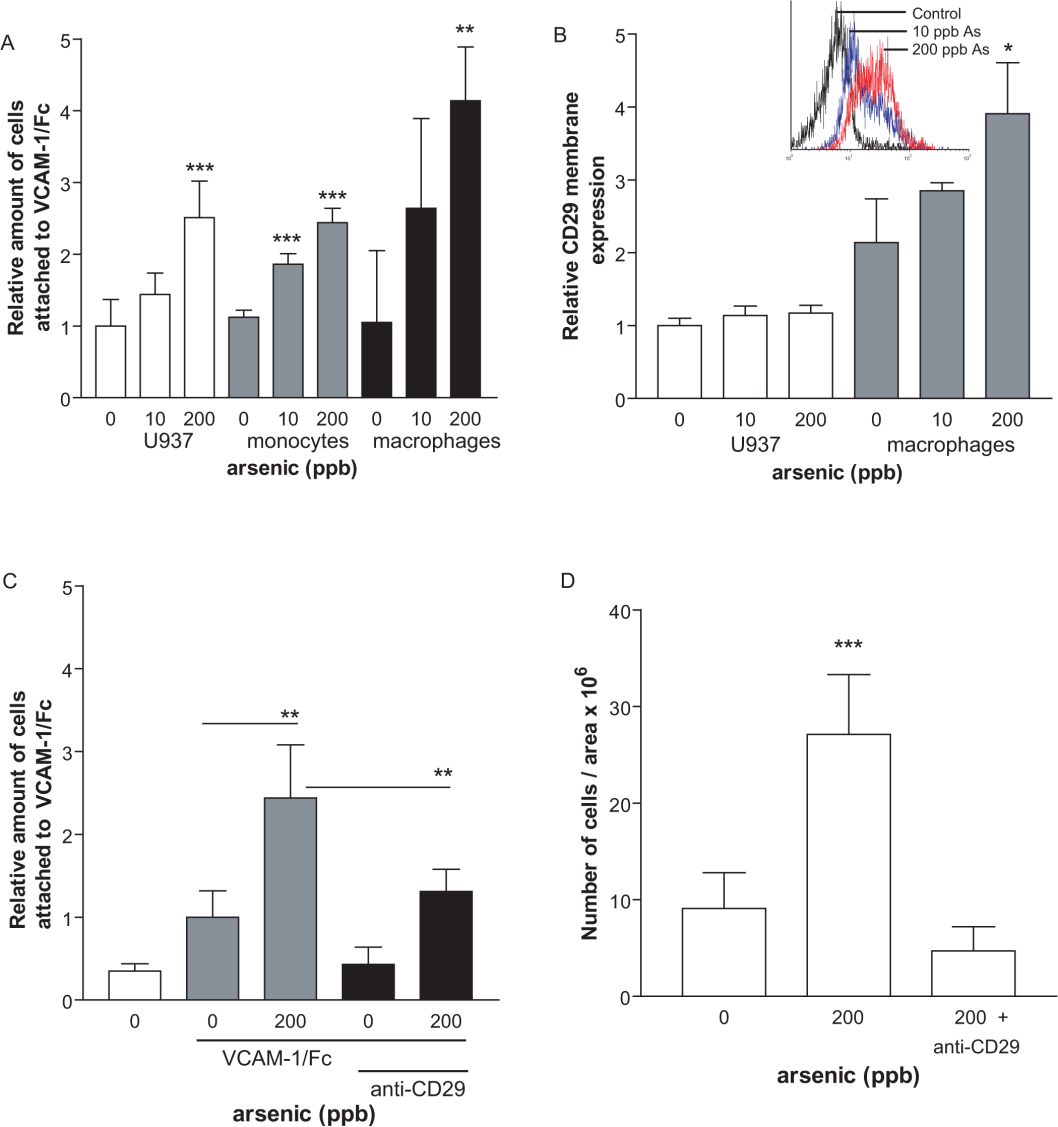
Arsenic increases adhesion of mononuclear cells via increased CD29 binding to VCAM-1. U937 or human PBMC cells (1000 cells/ml) were exposed to arsenic for 72h (0, 10 or 200 ppb) (A). Cells were fluorescently-labelled and incubated on VCAM-1/Fc coated plates. The non-adherent cells were washed away, and adherent fluorescent cells were counted. In B, cellular surface CD29 antigens were detected by flow-cytometry using anti-CD29 antibody. (C-D) CD29 blocking antibody was added to *in vitro* U937 binding assays to VCAM-1/Fc (C) or to *ex vivo* organ cultures with primary mononuclear cells (D). Values are expressed as mean ± S.D., n ≥ 3. * p < 0.05: **: p < 0.01; ***: p < 0.001, compared to unexposed controls.

By binding to VCAM-1, VLA-4 mediates the attachment of circulating cells to the activated vascular endothelium, favoring monocyte migration into the subendothelial space. VLA-4 is composed of CD49d (α4) and CD29 (β1) integrins. Interaction of monocyte VLA-4 with VCAM-1 occurs via inducible interactions between CD49d and CD29, producing changes in affinity (structural) or avidity (number) [37]. We tested whether arsenic exposure of monocytes could increase binding to immobilized VCAM-1. We observed that both human U937 monocytic cells and primary human monocytes adhered more to the VCAM-1/Fc chimera when exposed to arsenic for 72 h, as compared to the non-exposed control (Fig 3A). Furthermore, the presence of arsenic during the monocyte differentiation into macrophages also resulted in greater adhesion to VCAM-1 (Fig 3A). CD29 is constitutively expressed on monocytes [38], but upon activation, adopts an active conformation and its CD49a binding sites (the HUTS epitopes) become available for the antibody. Active-CD29 expression was slightly, but significantly, increased on human primary macrophages, but not monocytes, following arsenic exposure (Fig 3B; *: p < 0.05). We confirmed that CD29 was responsible for arsenic-increased binding to VCAM-1 by the addition of an anti-CD29 blocking antibody. Our results show a significant inhibition of U937 cell adhesion to VCAM-1 when blocking CD29 (Fig 3C; **: p < 0.01). We observed that the CD29 blocking antibody also prevented arsenic-induced binding of leukocytes to vascular endothelium in our *ex vivo* organ culture model (Fig 3D), supporting an important role for the VCAM-1/CD29 interaction in mediating the initial monocyte/endothelial cell interaction.

### Antioxidants can block binding of monocytes to VCAM-1 *in vitro* and *in vivo*

**Fig 4:**
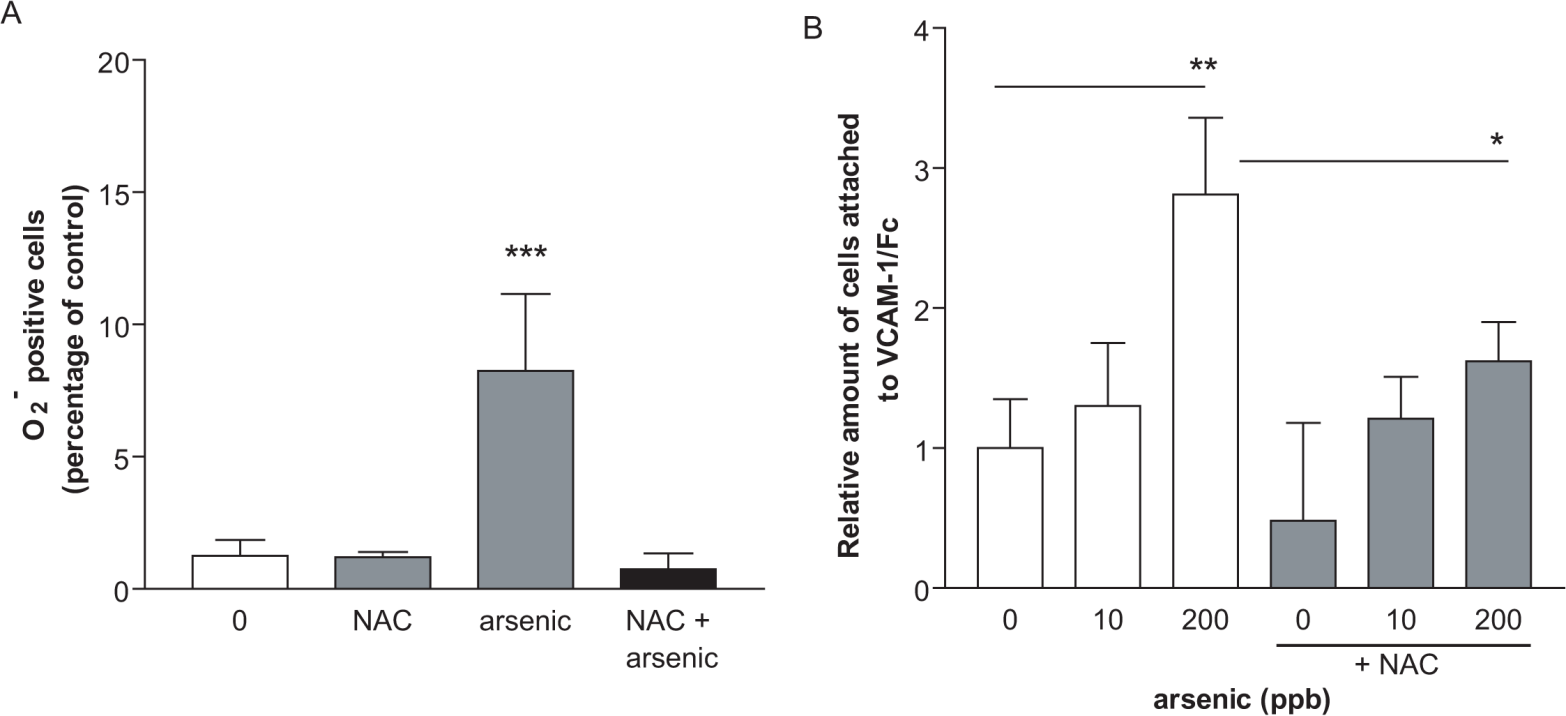
Arsenic-induced monocyte adhesion to VCAM-1 is prevented by antioxidant *in vitro*. U937 cells (1000 cells/ml) were pretreated for 1 h with NAC (1 mM) and then exposed to arsenic for 3h (0, 10, 50 or 200 ppb). Cells were stained with HEt and staining detected by flow cytometry (A). Alternatively, cells were fluorescently-labelled with orange tracker and incubated on VCAM-1/Fc coated plates (B). The non-adherent cells were washed away, and the adherent fluorescent cells were counted. Values are expressed as mean ± S.D., n ≥ 3. * p < 0.05: **: p < 0.01; ***: p < 0.001, compared to unexposed controls.

Arsenic is a potent inducer of reactive oxygen species (ROS) and many of the effects of arsenic have been attributed to ROS production [14,15]. In addition, VCAM-1 expression and signaling is known to be regulated through ROS [19,39]. Thus, we studied the role of arsenic-induced ROS in our models using NAC [40]. NAC increases the intracellular glutathione (GSH) pool, which can itself act as an antioxidant or can bind arsenic to increase its export [40]. First, we demonstrated that arsenic-increased ROS can be prevented *in vitro* by pre-treating U937 cells with NAC (Fig 4A). Second, we tested whether the increased binding of U937 cells to VCAM-1/Fc was dependent upon ROS. U937 cells were pre-treated for 1 hour with NAC, followed by 3 hours with arsenic before binding to VCAM-1/Fc was assessed. NAC significantly inhibited U937 cell binding to VCAM-1 (Fig 4B; *: p < 0.05), indicating that ROS mediate arsenic-increased adhesion of mononuclear cells to VCAM-1.

**Fig 5:**
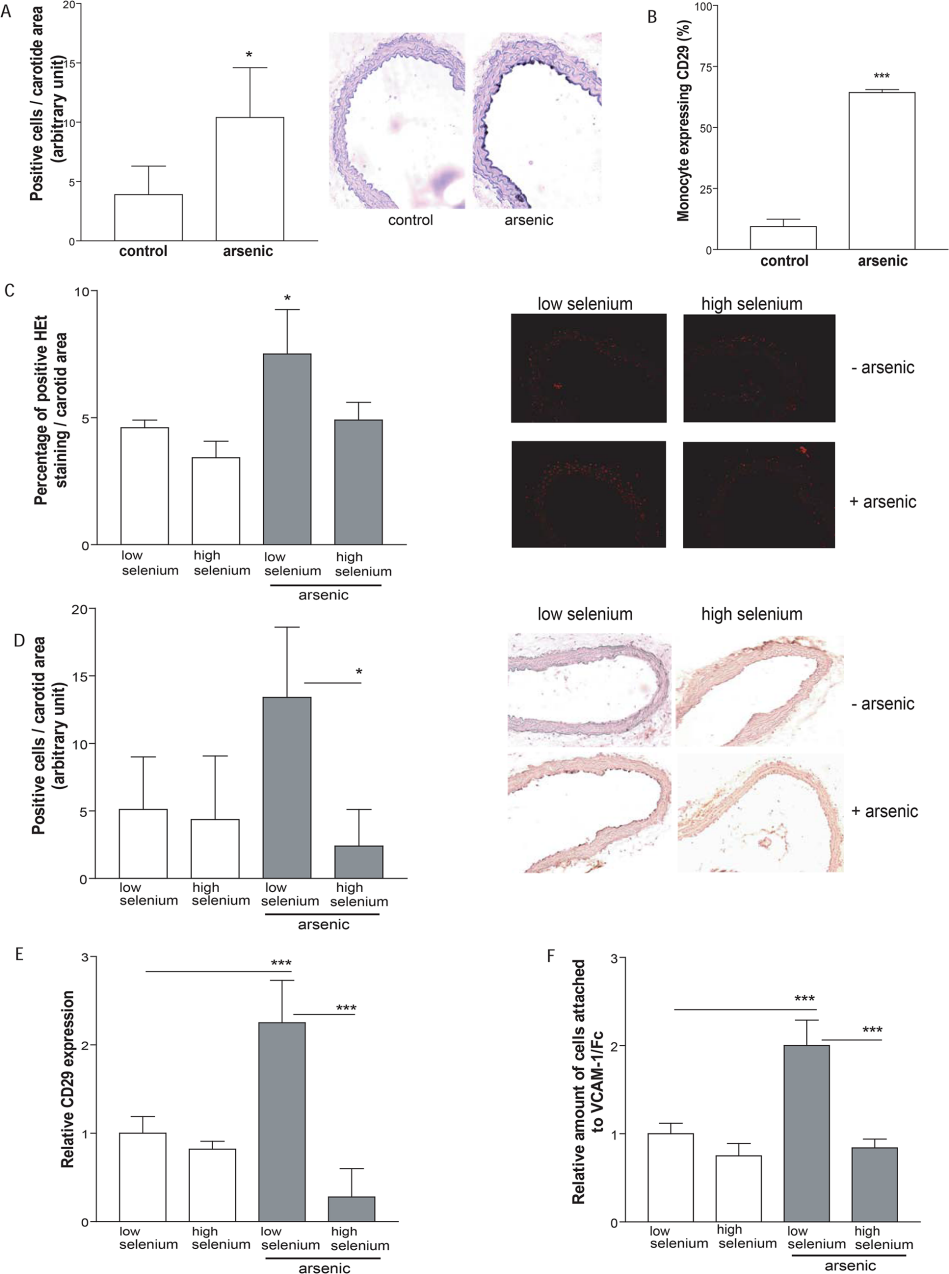
Arsenic increases adhesion molecule expression *in vivo*, which can be prevented by addition of high selenium diet. (A-B) In order to evaluate *in vivo* effects of arsenic, five-week-old male apoE^-/-^ mice fed normal rodent diet were exposed to arsenic (200 ppb) for 13 weeks or maintained on tap water. Carotids were stained for VCAM-1 (A) and whole blood was collected and CD29 expression was detected using flow-cytometry (B). (C-F) In order to evaluate ROS involvement in arsenic-induced atherosclerosis, five-week-old male apoE^-/-^ mice were exposed to arsenic (200 ppb) for 13 weeks or maintained on tap water. Mice were fed with low selenium or high selenium chow. Carotids were stained for ROS (C), or VCAM-1 (D). Blood was collected and CD29 expression was detected using flow-cytometry (E), or cells were fluorescently-labelled and incubated on VCAM-1/Fc coated plates (F). Values are expressed as mean ± S.D., n ≥ 3. * p < 0.05: **: p < 0.01; ***: p < 0.001, compared to unexposed controls.

To extend our *in vitro* findings, we utilized our *in vivo* model of arsenic-enhanced atherosclerosis to determine whether arsenic exposure correlated with increased adhesion molecule expression. Thus, we compared the expression of VCAM-1 on the endothelium of carotid arteries from tap water and arsenic-exposed, apoE^-/-^ mice. Indeed, endothelium from arsenic-exposed mice expressed significantly more VCAM-1 than the control group (Fig 5A). In addition, circulating monocytes of apoE^-/-^ mice exposed to arsenic expressed significantly more active-CD29 than their control counterparts (Fig 5B).

Finally, to evaluate the contribution of arsenic-induced ROS adhesion molecule expression and function *in vivo*, we co-exposed apoE^-/-^ mice to arsenic along with the antioxidant selenium. We chose to utilize this antioxidant, because we could incorporate variable levels in the animal chow (high and low selenium diet), in order to avoid interactions between the antioxidant and arsenic in the drinking water. We first confirmed that high levels of selenium prevented arsenic-induced ROS *in situ* in the carotids when compared to arsenic-exposed mice on a low selenium diet or unexposed mice (Fig 5C; *: p < 0.05). Arsenic-enhanced VCAM-1 staining was prevented when the animals were fed a diet-containing a high level of the antioxidant selenium (Fig 5D). Interestingly, active-CD29 levels on circulating monocytes were increased 2-fold in arsenic-exposed mice on low selenium diet, but this was abrogated in mice receiving a high selenium diet (Fig 5E). Elevated levels of active-CD29 correspondingly resulted in increased adhesion of circulating monocytes, when monocytes were removed from exposed mice and cultured on VCAM-1/Fc (Fig 5F). Together, these data suggest that arsenic-induced ROS likely plays a role in the increased endothelial VCAM-1 and mononuclear cell active-CD29 expression, which favors pro-atherogenic adhesion.

## Discussion

Despite compelling evidence that links environmental arsenic exposure to an increased risk of atherosclerosis [6,7,8], the mechanisms by which arsenic enhances atherosclerosis remain to be fully established. We hypothesized that it may increase pro-atherogenic cellular interactions important in the initial phases of the pathology. In this study, we demonstrate that arsenic exposure specifically enhances mononuclear-endothelium interactions *in vitro* and *ex vivo*. Arsenic increases VCAM-1 and CD29 adhesion molecules to increase monocyte binding, which can be prevented *in vitro* and *in vivo* by antioxidants. In fact, we observed that arsenic exposure specifically enhanced monocyte attachment to endothelial cells (Fig 1), but failed to induce platelet activation and aggregation with leukocytes (Fig 2). In our apoE^-/-^ mouse model of atherosclerosis, arsenic increased vascular expression of VCAM-1 and monocyte expression of active CD29 (Fig 5). Interestingly, arsenic-induced VCAM-1 expression and monocyte adhesion could be prevented by co-exposure with the dietary antioxidant selenium (Fig 5). Thus, we believe that in part, arsenic-induced pro-atherogenic mechanisms are linked to enhanced circulating monocyte adhesion to the endothelium through generation of ROS.

Several of the mechanisms implicated in arsenic-enhanced atherosclerosis are associated with endothelial activation, such as production of inflammatory molecules [11,23] and ROS [14,15]. The first adhesion molecule expressed upon endothelial cell activation is VCAM-1, which is virtually absent on the vasculature prior to activation [41]. VCAM-1 expression is uniquely upregulated upon atherosclerotic stimuli [42], and exacerbation of cellular recruitment to VCAM-1 contributes to atherosclerosis [43]. ICAM-1 (intercellular adhesion molecule-1) is also expressed upon endothelial activation [41,42,44] but, VCAM-1 is the major player in the establishment of the nascent lesion [45]. Hyperlipidemic Ldlr^-/-^ mice lacking the VCAM-1 extracellular domain displayed decreased plaque formation compared to Ldlr^-/-^ mice with wild-type VCAM-1, while deletion of ICAM-1 did not alter the early plaque formation [45]. Both human and murine atherosclerotic vascular lesions express VCAM-1, which correlates with the extent of exposure to the pro-atherogenic stimuli [46]. Thus, we focused our investigations on VCAM-1, and found that atherogenic concentrations of arsenic [9,47] induced vascular endothelial VCAM-1 expression at lesion-prone sites in apoE^-/-^ mice model of atherosclerosis (Fig 5A). Interestingly, this supports the positive association observed between arsenic exposure and increased human soluble VCAM-1 concentration in the plasma [48,49], which is known to increase with endothelium activation in atherosclerosis [50].

Our *in vitro* and *in vivo* data support the hypothesis that increased VCAM-1/integrin adhesion is dependent upon ROS. Antioxidants inhibited arsenic-induced monocyte binding to VCAM-1 *in vitro* (Fig 4). A high selenium diet decreased VCAM-1 expression on vascular endothelial cells and decreased active-CD29 expression on monocytes *in vivo*, associated with decreased monocyte binding capacity to VCAM-1 (Fig 5). Selenium is an essential micronutrient, and acts as an antioxidant through its action as a co-factor for enzymes, such as GSH peroxidase and thioredoxin reductase [51]. It also binds arsenic to form the seleno-bis(S-glutathionyl) arsinium ion, which enhances excretion through the hepatobiliary system [52]. Thus, selenium co-exposure will increase not only the total antioxidant capacity, but also the clearance of arsenic, which both should prevent ROS damage.

The role of ROS in mediating increased adhesion may be multi-fold. ROS may play a role in VCAM-1 expression via NFκB activation [53]. Interestingly, arsenic induces NFκB [54], which might be responsible for the observed arsenic-increased VCAM-1 expression in our apoE^-/-^ mouse model (Fig 5). The binding of VCAM-1 to the VLA-4 integrin rapidly activates the production of low concentrations of H_2_O_2_ in the endothelial cell [19] that signals to activate matrix metalloproteinases-2 and -9 (MMP-2/9) [39]. MMPs degrade extracellular matrix and cleave endothelial cell junctions to allow monocyte diapedesis [51,55]. We previously observed that arsenic exposure slightly, but not significantly, induces MMP activity within the apoE^-/-^ atheroma [47]. Thus, arsenic-induced ROS could promote the monocyte transmigration through the endothelial cell layer by enhancing VCAM-1 expression even in the absence of a supplementary effect on MMPs.

We demonstrated here that arsenic exposure enhances monocyte interaction of VLA-4 with endothelial adhesion molecule VCAM-1 (Fig 1). Increased binding might be the critical step for arsenic-induced plaque formation *in vivo*, because circulating monocyte firm adhesion to the endothelium is required for atherosclerosis formation [43]. Inhibition of VLA-4 with blocking antibodies has been shown to prevent monocyte adhesion in apoE^-/-^ fed high fat diet [56,57]. We confirmed that utilization of blocking antibody targeting CD29 specifically prevents arsenic-induced monocytes adhesion to VCAM-1, suggesting the biological importance of this integrin in arsenic-induced atherosclerosis. Although present on lymphocytes, VLA-4 integrin is responsible for the specific monocytic binding to VCAM-1 [42]. Interestingly, arsenic-exposed apoE^-/-^ mice displayed increased monocytic active-CD29 expression, which correlates with increased vascular VCAM-1 expression (Fig 5). This might suggest that arsenic is able to trigger activated VLA-4 conformation itself. This is even more likely when we consider that in order to have maximal binding *in vitro* or *ex vivo*, both mononuclear and endothelial cells need to be exposed to arsenic (Fig 1). While CD29 is constitutively expressed in monocytes [38], the regulation of its active form is proposed to arise from either β2 chain integrin engagement [58], via modification in CD49d α4 transcription level [59] or through ROS-dependent mechanisms [60]. We had assessed CD49d α4 mRNA expression in macrophages with and without arsenic and saw no change (unpublished data), thus we focused on arsenic-mediated ROS effects. Interestingly, the antioxidant NAC was efficient in preventing ionizing radiation-induced CD29 β1 membrane expression in murine RAW.267 macrophages [60]. The authors proposed that it might explain atherosclerosis formation observed in cancer patients receiving ionizing radiation. Further investigation is needed to understand arsenic-induced regulation of CD29 integrin, but interestingly, we observed that *in vivo* arsenic-enhanced CD29 expression is prevented when apoE^-/-^ mice are co-exposed to selenium, which suggests that arsenic may control CD29 regulation through ROS production (Fig 5).

Although we found that arsenic enhanced monocyte/endothelial cell adhesion, we found no evidence, from either wild-type or atherogenic mice, of increased platelet activation or platelet/leukocyte aggregates, all of which are involved in plaque initiation [35,61,62]. This contrasts with previous reports that arsenic induces platelet activation and aggregation *in vit*ro and *in vivo*, resulting in enhanced arterial thrombosis [33]. However, we utilized much lower concentrations of arsenic [up to a maximum of 200 ppb sodium arsenite (2.6 μM arsenic) for 5 min], whereas previous reports indicate that platelets require much higher concentrations of arsenic (above 10 μM) to reach activation at significantly longer exposure periods. Furthermore, we did not expose platelets to an inhibitor of platelet aggregation prior to arsenic exposure, which also might explain part of the discrepancies. To confirm our data *in vivo*, we exposed apoE^-/-^ mice to pro-atherogenic arsenic concentration, and assessed circulating levels of CCL5, a major cytokine secreted upon platelet activation [63] that contributes to mononuclear cell accumulation at the lesion site in this model [64]. However, arsenic exposure did not alter CCL5 plasma concentrations (Fig 2). Furthermore, we found no evidence of increased circulating platelet/leukocyte aggregates over one month exposure to arsenic (Fig 2), suggesting limited participation of platelets in arsenic-increased atherosclerosis. This highlights the specificity of increased monocyte/endothelial cell adhesion following arsenic exposure, rather than implicating a general increase in intercellular interactions.

Together, our data suggest that arsenic promotes monocyte adhesion to endothelial cells, providing a possible mechanism for the effect of arsenic on atherosclerosis. Furthermore, our studies indicate that these early events in atherosclerosis induced by arsenic can be prevented by antioxidants. Thus, prevention of atherosclerosis may be possible in high risk populations exposed to arsenic.

## Supporting Information

S1 Fig Arsenic does not increase CCL2 circulating levels. Circulating CCL2 levels were measured in apoE^-/-^ male mice that received tap water or that were exposed to 200 ppb arsenic for 8 or 13 weeks using an immunoassay kit (multiplex bead-based) on a Bio-Plex 200 (Bio-Rad Laboratories). Each sample (n = 4) was analyzed in duplicate (technical replicate). Values are expressed as mean ± S.D.
(EPS)
